# The implementation of the nationwide out-of-hours phone number 1733 in Belgium: analysis of efficiency and safety

**DOI:** 10.1017/S1463423621000098

**Published:** 2021-03-15

**Authors:** Birgitte Schoenmakers, Lukas Delmeiren, Sjors Pietermans, Marco Janssens, Chris Van Der Mullen, Marc Sabbe

**Affiliations:** Department of Public Health and Primary Care, KU Leuven, Leuven, Belgium

**Keywords:** after-hours care, family physicians, primary health care, telephone, triage methods

## Abstract

**Background:**

Belgium has a problem with inappropriate use of emergency services. The government installed the number 1733 for out-of-hours care. Through a dry run test, we learned that 30% of all calls were allocated to the protocol ‘unclear problem’. In only 11.9% of all cases, there was an unclear problem.

**Methods:**

The study aimed to determine whether the adjusted protocol ‘unwell for no clear reason’ led to a safer and more efficient referral and to evaluate the efficiency and safety of the primary care protocols (PCPs). The study ran in cross-sectional design involving patients, General Practitioner Cooperatives and telephone operators. A random sample of calls to 1733 and patient referrals were assessed on efficiency and safety.

**Results:**

During 6 months in 2018, 11 622 calls to 1733 were registered. Seven hundred fifty-six of them were allocated to ‘unwell for no clear reason’, and a random sample of 180 calls was audited. To evaluate the PCPs, 202 calls were audited. The efficiency and safety of the protocol ‘unwell for no clear reason’ improved, and safety levels for under- and over-triage were not exceeded. The GP’s judged that 9/10 of all patient encounters were correctly referred.

**Conclusion:**

This study demonstrated that the 1733-telephone triage system for out-of-hours care is successful if protocols, flow charts and emergency levels are well defined, monitored and operators are trained.

## Introduction

In Belgium, there is problem with the inappropriate use of emergency services (Brasseur *et al.*, 2019a; Philips *et al.*, 2010a). In 2012, we registered 290 emergency contacts per 1000 inhabitants, which is far above the average in comparable nations (Carret *et al.*, 2009; Philips *et al.*, 2014; Uscher-Pines *et al.*, 2013). Besides, 71% of the patients are self-referrers. Since 2003, 80 general practitioner cooperatives (GPCs) were established offering out-of-hours care in a primary care context. Against all expectations, the number of emergency contacts did not decrease (Philips *et al.*, 2010b). The opening hours of a GPC are restricted, there are no formal agreements between GPC and ED (emergency department) and, above all, patients are more confident with the service of an ED.

A nationwide study demonstrated that there is a need to develop an effective triage system improving the out-of-hours referring process (Van den Heede *et al.*, 2016). From a review, we also know that the introduction of telephone triage systems is a promising intervention to reduce inappropriate ED visits (Van den Heede & Van de Voorde, 2016).

The overuse of the ED is also encouraged by free access (Carret *et al.*, 2007). This inappropriate use of the ED undermines the functioning of GPCs for out-of-hours care (O’Kelly *et al.*, 2010; Huibers *et al.*, 2013; Philips *et al.*, 2010b). The government installed the nationwide number 1733 for unplanned, non-live-threatening out-of-hours care. An operator directs the caller to the appropriate level of care: three levels of ambulance intervention, urgent or planned referral to out-of-hours primary care services or to planned care (https://www.health.belgium.be/en/health/need-call-doctor-call-1733). These operators are trained to follow digital protocols. These protocols are the result of a collaboration between academic partners, the Federal Public Service Health, experts in general practice and in emergency medicine.

To evaluate the efficiency and safety of the use of the initial protocols, a dry run test phase was implemented (Van der Mullen *et al.*, 2017). All patients were asked to call the 1733 emergency number in the presence of a researcher. The operator interrogated the patient following a flow chart and referred according to the chosen protocol to the appropriate care level. In 30% of all calls, the operator assigned the call to the protocol ‘unclear problem’. In 11.9% of all these cases, there was indeed an unclear problem (eg, communication, multiple complaints). In all other cases, there was no appropriate protocol available or there was a problem on operator level.

The initial protocols were adjusted and completed with specific primary care protocols (PCP) (attachment 1). The operators received instructions and dry run training by GP- and ED-experts to improve the use of the new protocols. The protocol ‘unclear problem’ was renamed to ‘unwell for no clear reason’. The Belgian Counsel for Urgent Healthcare approved this final version of the protocols.

In international literature, there is a lack of research on the implementation of a central, nationwide number for unplanned out-of-hours care. Since many health care systems are confronted with inefficient patient flows in unplanned or emergency care, an implementation research on this topic might contribute to improvement of this particular type of care (Ghazali *et al.*, 2019; Young *et al.*, 1996).

Therefore, the aim of this research was to determine whether the adjusted protocol ‘unwell for no clear reason’ led to a safer and more efficient referral of calls to the appropriate care level and to evaluate the efficiency and safety of the specific PCPs.

## Methods

### Design

The study was performed in a cross-sectional design. The earlier protocol ‘unclear problem’ was adjusted and renamed to ‘unwell for no clear reason’. The operator chooses this protocol when the caller reports complaints that do not fit into any other protocol.

A protocol is considered as efficient when it leads with the appropriate care level: three levels of ambulance intervention, urgent or planned referral to out-of-hours primary care services or to planned care. A protocol is considered safe when the rate of under-triage is <15%. Both decisions on efficiency and safety were the result of a consensus between experts, the Intermediary Report Project 1733, guidelines and national research reports (Wheeler *et al.*, 2015; Van der Mullen *et al.*, 2017; Van den Heede & Van de Voorde, 2016; Uscher-Pines *et al.*, 2013).

The 1733 operator follows a flow chart, instructed by the appropriate protocol, to question and to refer the caller. Each flow chart starts with a set of medical terms. Each protocol integrates different levels of severity leading to a particular level of care.

To determine whether the adjusted protocol ‘unwell for no clear reason’ leads to a safer and more efficient referral, a set of calls was audited by two researchers. A computerized random selection was made of all calls recognized as ‘unwell for no clear reason’ between May 4 and June 26, 2018. For the collection of data, a template inventorying time of day, patient history, protocol choice and level of care structured the assessment. The researchers individually assessed the calls, marked their protocol of choice and motivated this choice: correct protocol, missing protocol, fail to recognize keyword, fail of the operator, communication problem and impossible to refer. At this stage, the researchers were not in contact with each other.

The data were qualitatively analyzed according to the 'Grounded Theory approach'. For the qualitative part, the researchers (*n* = 5) discussed the results of the assessment of calls in different rounds. In case of disagreement with the operators, the call was labeled as ‘discordant’. In case the researchers did not reach consensus, the call was presented to the advisory group. If the operator initially referred to a lower care level, the call was labeled as ‘under-triage’ (with the number of levels below). If the operator initially referred to a higher care level, the call was labeled as over-triage.

In the second research question, the efficiency and safety of the specific PCPs were studied following the same procedure as described above. For each of the 18 PCPs, 15 calls were randomly selected by randomization software from January 1 until June 30, 2018. All patients referred to these protocols were assessed by the GP on duty (Weekends July 27–30, 2018 and February 8–11, 2019). The assessment took place when the patient presented at the GPC and before the consult started. The GP asked the patients the reason for encounter and then selected the level of care. This option was compared to the option the operator picked. The outcome of this assessment did not affect the provided care.

Efficiency of referral by the operator was expressed as the level of concordance between the operators’ and the GPs’ referrals. Safety was measured by the occurrence and the level of under-triage.

### Ethical approval

This study was approved per research question by the Medical Ethical Committee of the University Hospitals of the KU Leuven (041C55FA505A80, 04324FEAA15C80 and 047066BAC85E80).

## Results

### Efficiency and safety of ‘unwell for no clear reason’

For the period of January 2018 to June 30, 2018, 11 622 calls were registered. 756 (6.6%) of these calls were allocated to the protocol ‘unwell for no clear reason’ of which a random sample of 180 calls was audited. Sixteen calls were excluded because of technical errors. In total, 164 calls were included.

The researchers assigned the label ‘fail to recognize keyword’ to 51 calls (31%), the label ‘correct protocol’ to 44 (27%) calls and the label ‘missing protocol’ to 37 (23%) calls. They assigned the label ‘fail of the operator’ to 23 (14%) calls. Nine (5%) calls were labeled as ‘communication problem’. No call was labeled as ‘impossible to refer’ (Table 1).

**Table 1.** Efficiency and safety of ‘Unwell for no clear reason’

*Variable*		*Number of calls (%)*		
Included calls		164/756 (22%)		
Time of day		Morning = 60 (37%) calls Afternoon = 45 (26.8%) calls Evening 44 = (27%) calls Night 15 (9.9%) = calls		
Label after assessment of content of call		fail to recognize keyword’ = 51 (31%) calls correct protocol = 44 (27%) calls missing protocol = 37 (23%) calls fail of the operator = 23 (14%) calls, communication problem = 9 (5%)		
Label after assessment of efficiency of allocation to protocol		unwell for no clear reason = 104 (64%) calls dizziness = 8 (5%) skin problems = 6 (3%) airway problems = 5 (3%) other calls (*n* = 41/25%) less than 5 time to another protocol		

Assessment of safety of referral				
	Care level operator		Care level researcher	Number (%)
Under-triage, *n* = 17	GP urgent		Ambulance	1 (0.6%)
	Out of hours primary care services		GP urgent	13 (8%)
	Out of hours primary care services		Ambulance	2 (1%)
	Planned care		Out of hours primary care services	1 (0.6%)
Over-triage, *n* = 36	Ambulance		GP urgent	1 (0.6%)
	GP urgent		Out of hours primary care services	8 (5%)
	Out of hours primary care services		Planned care	27 (16.5%)

Discordance of care level when other protocol				
	Care level operator		Care level researcher	
Under-triage, *n* = 13	Out of hours primary care services		GP urgent	9 (5.5%)
	Out of hours primary care services		Ambulance	2 (1%)
	GP urgent		Ambulance	2 (1%)
Over-triage, *n* = 12	GP urgent		Out of hours primary care services	5 (3%)
	Out of hours primary care services		Planned care	7 (4%)

When assessing the efficiency of allocation to a protocol, 104 (64%) calls were labeled as ‘unwell for no clear reason’. Eight calls (5%) were allocated to the protocol ‘dizziness’, six calls to the protocol ‘skin problems’ and five (3%) calls to ‘airway problems’ (Table 1).

The operator referred 135 (82%) to the out-of-hours primary care services and 23 (14%) calls were urgently referred. Planned care was advised in 4 (3%) calls. Two (1%) calls were referred to ‘ambulance intervention’.

When assessing the safety of referral, the researchers referred 111 (68%) calls to the same level of care as the operator. 36 (22% of total) of all calls were recognized as ‘over triage’ and 17 (10% of total) as ‘under triage’ (Table 1).

The allocation to an inappropriate protocol led in 25 (15%) calls to a discordance of care level between the operator and the researcher. In 12 calls, the referral was labeled as ‘over-triage’ and 13 calls were recognized as ‘under triage’ (Table 1).

### The efficiency and safety of (referral to) the specific PCPs

For each of the 18 PCPs, 15 calls were randomly selected for assessment (Table 2). For seven protocols, <15 calls were available. The researchers assessed 202 calls. The researchers assessed 126 (62%) calls as ‘correct protocol’, 13 (6%) as ‘missing protocol’, 48 (24%) as ‘fail to recognize keyword’, 12 (6%) as ‘fail of the operator’ and three (2%) as ‘communication problem’. To determine the efficiency of the PCP, the researchers assessed the allocation to a protocol. 76 (37%) calls were allocated to another protocol: 16 (8%) calls were allocated to ‘unwell for no clear reason’, 10 (5%) to ‘child with fever’, 6 (3%) to ‘wounds’, 5 (2.5%) to ‘abdominal pain’ and 4 (2.5%) to both ‘ear-nose-throat’ and ‘allergic reaction’.

**Table 2.** The efficiency and safety of (referral to) the specific primary care protocols

*Variable*		*Number of calls (%)*		
Included calls		202		
Time of day		Morning 75 (37%) Afternoon 65 (32%) Evening 49 (24%) Night 13 (7%)		
Label after assessment of content of call		correct protocol = 126 (62%) calls missing protocol = 13 (6%) calls fail of the operator = 12 (6%) fail to recognize keyword = 48 (24%) calls communication problem = 3 (2%) calls		
Label after assessment of efficiency of allocation to protocol		unwell for no clear reason = 16 (8%) calls child with fever =10 (5%) calls wounds = 6 (3%) calls abdominal pain =5 (2.5%) calls ear-nose-throat’ or allergic reaction = 4 (2.5%) calls other calls (*n* = 31/15%) less than 5 time to another protocol		

Assessment of safety of referral				
	Care level operator		Care level researcher	Number (%)
Under-triage, *n* = 22	Out of hours primary care services		GP urgent	17 (8.5%)
	Out of hours primary care services		Ambulance	5 (2.5%)
Over-triage, *n* = 24	Out of hours primary care services		Planned care	18 (9.0%)
	GP urgent		Out of hours primary care services	3 (1.5%)
	Ambulance		GP urgent	2 (1.0%)
	Ambulance		Out of hours primary care services	1 (0.5%)

Discordance of care level when other protocol				
	Care level operator		Care level researcher	Number (%)
Under-triage, *n* = 11	Out of hours primary care services		GP urgent	9 (5%)
	Ambulance		GP urgent	2 (1%)
Over-triage, *n *= 7	Out of hours primary care services		Planned care	6 (3%)
	GP urgent		Out of hours primary care services	1 (0.5%)

To determine the safety of referral to the PCP, the researchers assessed the allocation to the level of care (Table 2). 175 calls (87%) were allocated to ‘out of hours primary care services’, 19 calls (9%) to ‘urgent out of hours primary care services’, 7 calls (3%) to ‘ambulance’ and 1 (1%) call to ‘planned care’. The researchers referred 46 (23%) calls to another care level: in 22 (11%) calls, there was under-triage and in 24 (12%) over-triage. The researchers disagreed about the level of care in 10 calls. These calls were audited and discussed until consensus: two calls remained undetermined due to a language issue and missing administration. The researchers labeled 100 (50%) calls as ‘correct protocol and correct care level’ and 27 (13%) calls as ‘inappropriate protocol and correct care level’. 57 (28%) calls were labeled as ‘incorrect protocol’ but correct care level. In 18 (8%) cases, the researchers labeled a call as ‘inappropriate protocol and inappropriate care level’. Nine (6%) calls were referred to a lower care level than appropriate and seven (3.5%) to a higher level.

To determine the safety of referral to a care level using the PCP, 325 allocated patients received an assessment by the GP (Table 3). Seven assessments were incomplete, and 68 assessments were completed after the consultation. In total, 250 assessments were included.

**Table 3.** Discordance of allocation to care level between operator and GP on duty

Triage			Summer	Winter	Total
Correct			99 (97.1%)	137 (92.6%)	236 (94.4%)
Not correct			3 (2.9%)	11 (7.4%)	14 (5.6%)
Over-triage	Care level operator	Care level GP			
	Out of hours primary care services	Planned care	1	7	8
	Urgent home visit	Regular home visit	0	3	3
	Home visit	Planned care	1	0	1
Under-triage	Home visit	Urgent home visit	1	0	1
	Urgent home visit	Urgent Ambulance	0	1	1

In the summer weekend, the following reasons for encounter were withheld: 24 (10.5%) wound/skin problem, 15 (15%) stomach-intestines, 10 (10%) ear-nose-throat, 7 (7%) uro-genital, 6 (6%) allergic reaction/insect bite and 40 (39%) all other problems <2% prevalent. In the winter weekend, reason for encounter was in 45 cases (32%) ear-nose-throat, 19 (12%) lung, 15 (10%) stomach-intestines, 14 (9%) unwell for no clear reason, 10 (7%) uro-genital and 45 (30%) other reasons.

Of all patients presenting at the guard post, the operator allocated 181 (73%) cases to a regular consultation, 43 (19%) to regular home visit and 26 (8%) to urgent home visit. In summer versus winter 6 (5%), respectively, 13 (9%) cases concerned an urgent home visit, 25 (25%), respectively, 22 (15%) a regular home visit, 71 (70%), respectively, 113 (76%) a regular consultation.

After assessment by the GP, 236 (94.4%) calls were correctly referred. In two (0.8%) cases, the patients needed referring to a higher level of care and in 12 (5%) cases to a lower level of care.

## Discussion

The efficiency and safety of the protocol ‘unwell for no clear reason’ improved after adjusting the protocols and adding PCPs. The number of calls referred to this protocol decreased as compared to the first trial. The concordance rate between allocation of a call to this protocol by operator and researcher was 2/3, which was higher than in the test phase. The rate of under-triage by allocation to ‘unwell for no clear reason’ was 1/10. By implementing new PCPs, there was a halving of allocation to ‘missing protocol’. The concordance of allocation to the new PCPs by operator and researcher was 2/3, but the safety levels for under- and over-triage were not exceeded. The GP’s on duty judged that over 9/10 of all patient encounters were correctly allocated. In <1% of all referrals, the GP marked an under-triage.

In one-third of the referrals to ‘unwell for no clear reason’, the call was labeled as ‘fail to recognize key word’. This rate was higher than that in the test phase and could be explained by the lack of experience with the new protocols. Training of the operators is therefore very important (Hansen & Hunskaar, 2011; Huibers *et al.*, 2011).

By improving the PCPs, there was a manifest decrease of the number of calls assigned to ‘missing protocol’. When assessing the allocation of calls to the protocol ‘unwell for no clear reason’, the researchers now lacked the protocols ‘fever adult’, ‘breast problems’ and ‘hypertension’. By developing and implementing these protocols, the number of allocations to ‘unwell for no clear reason’ could further decrease (Smits *et al.*, 2016; Brasseur *et al.*, 2019b). In addition, researchers assessed half of the allocations to the PCP as correct for protocol and care level, and this rate could benefit from adding new PCP.

The implementation of PCP seemed successful since operators allocated more than 9/10 of all calls to the GP guard post. Only 1% of all calls was referred to planned care. This number is low and probably due to a lack of experience with this newly introduced care level (Rutten *et al.*, 2017; Plat *et al.*, 2018; Morreel *et al.*, 2019).

The main reasons of discordance between operator and researcher in allocation to the PCP were a fail of the operator and a fail to recognize the keyword. This was particularly the case when the age of the caller was important to allocate to a protocol. Operators often failed to question the context and the state of emergency. Time, experience and training were lacking present. A digitized algorithm could support the operators (Smits *et al.*, 2016; Smits *et al.*, 2017; Brasseur *et al.*, 2019b). Operators were confronted with dissatisfied callers who expected to receive a medical advice (Giesen *et al.*, 2007; Huibers *et al.*, 2011; Huibers *et al.*, 2012). Public information campaigns are necessary to align the objectives of the 1733-system and the public expectations (Morreel *et al.*, 2019; Philips *et al.*, 2019). Strikingly, the operators only once opted for the care level ‘planned care’, and the researchers also rarely opted this care level. Operators are not familiar with this newly introduced care level and callers not used to referral to postponed care. Language barriers and missing keywords in protocols were also reasons for incorrect referral. Probably, this is a point of attention that needs evaluation on regular base (Huibers *et al.*, 2011; van der Straten *et al.*, 2012).

The rate of under- and over-triage was acceptable as appeared by re-listening the calls and by pre-consultation assessment at the guard post. Over-triage was more likely than under-triage and mainly due to referral to care level ‘out of hours primary care services’ instead of referral to ‘planned care’. Unlike over-triage, under-triage may result in an unsafe situation (Huibers *et al.*, 2011; Plat *et al.*, 2018; Brasseur *et al.*, 2019b). Under-triage was caused by referral to ‘out of hours primary care services’ instead of ‘urgent home visit’. In two cases, there was an unacceptable under-triage by referring the patient to ‘out of hours primary care services’ instead to ‘ambulance’.

The strength of this research lies in the fact that it started in a dry run phase and, after evaluation and adjustment, continued in a real-life setting. Second, the protocols are now fully used in the 1733 operating center. The researchers (GP’s) took part in the development and fine-tuning of the protocols during the first test phase of the implementation of the protocol.

An important limitation of the study is that the assessment of calls was based upon the competence of the researchers. Indeed, the researchers were GPs, while the majority of the operators do not have a medical background (Plat *et al.*, 2018). Although a thorough reviewing and revision of the results was performed, misinterpretations cannot be ruled out.

## Conclusion

This study demonstrated that the 1733-telephone triage system for out-of-hours care is successful if protocols, flow charts and emergency levels are well defined, continuously monitored and operators well trained. Further research should focus on the rate and conditions of under- and over-triage to improve the competences and skills of the operators. In practice, selection, education and training of operators are indispensable to guarantee safety and cost efficiency.

## References

[ref1] Brasseur E , Gilbert A , Servotte JC , Donneau AF , D’Orio V and Ghuysen A (2019a) Emergency department crowding: why do patients walk-in? Acta Clinica Belgica, 1–7. 10.1080/17843286.2019.1710040 31886742

[ref2] Brasseur E , Servotte JC , Donneau AF , Stipulante S , D’Orio V and Ghuysen A (2019b) Triage for out-of-hours primary care calls: a reliability study of a new French-language algorithm, the SALOMON rule. Scandinavian Journal of Primary Health Care 37, 227–232. 31033368 10.1080/02813432.2019.1608057 PMC6567030

[ref3] Carret ML , Fassa AC and Domingues MR (2009) Inappropriate use of emergency services: a systematic review of prevalence and associated factors. Cadernos de Saude Publica 25, 7–28. 19180283 10.1590/s0102-311x2009000100002

[ref4] Carret ML , Fassa AG and Kawachi I (2007) Demand for emergency health service: factors associated with inappropriate use. BMC Health Service Research 7, 131. 10.1186/1472-6963-7-131 PMC2034385 17705873

[ref5] Ghazali DA , Richard A , Chaudet A , Choquet C , Guericolas M and Casalino E (2019) Profile and motivation of patients consulting in emergency departments while not requiring such a level of care. International Journal of Environmental Research and Public Health 16(22), 4431. 10.3390/ijerph16224431 PMC6888183 31726697

[ref6] Giesen P , Ferwerda R , Tijssen R , Mokkink H , Drijver R , Van Den Bosch W and Grol R (2007) Safety of telephone triage in general practitioner cooperatives: do triage nurses correctly estimate urgency? Quality & Safety in Health Care 16, 181–184. 17545343 10.1136/qshc.2006.018846 PMC2465002

[ref7] Hansen EH and Hunskaar S (2011) Telephone triage by nurses in primary care out-of-hours services in Norway: an evaluation study based on written case scenarios. BMJ Quality & Safety 20, 390–396. 10.1136/bmjqs.2010.040824 PMC3088408 21262792

[ref8] Huibers L , Giesen P , Smits M , Mokkink H , Grol R and Wensing M (2012) Nurse telephone triage in Dutch out-of-hours primary care: the relation between history taking and urgency estimation. European Journal of Emergency Medicine 19, 309–315. 22008589 10.1097/MEJ.0b013e32834d3e67

[ref9] Huibers L , Smits M , Renaud V , Giesen P and Wensing M (2011) Safety of telephone triage in out-of-hours care: a systematic review. Scandinavian Journal of Primary Health Care 29, 198–209. 22126218 10.3109/02813432.2011.629150 PMC3308461

[ref10] Huibers L , Thijssen W , Koetsenruijter J , Giesen P , Grol R and Wensing M (2013) GP cooperative and emergency department: an exploration of patient flows. Journal of Evaluation in Clinical Practice 19, 243–249. 22304568 10.1111/j.1365-2753.2011.01806.x

[ref11] Morreel S , Philips H and Verhoeven V (2019) Self-triage at an urgent care collaboration with and without information campaign. Journal of Emergency Management 17, 511–516. 31903540 10.5055/jem.2019.0443

[ref12] O’kelly FD , Teljeur C , Carter I and Plunkett PK (2010) Impact of a GP cooperative on lower acuity emergency department attendances. Emergency Medicine Journal 27, 770–773. 20378743 10.1136/emj.2009.072686

[ref13] Philips H , Michiels B , Coenen S and Remmen R (2014) Reducing inappropriate A&E attendances. British Journal of General Practice 64, 70. 10.3399/bjgp14X677031 PMC3905416 24567592

[ref14] Philips H , Remmen R , De Paepe P , Buylaert W and Van Royen P (2010a) Out of hours care: a profile analysis of patients attending the emergency department and the general practitioner on call. BMC Family Practice 11, 88. 21078162 10.1186/1471-2296-11-88 PMC2998456

[ref15] Philips H , Remmen R , Van Royen P , Teblick M , Geudens L , Bronckaers M and Meeuwis H (2010b) What’s the effect of the implementation of general practitioner cooperatives on caseload? Prospective intervention study on primary and secondary care. BMC Health Services Research 10, 222. 20673342 10.1186/1472-6963-10-222 PMC2922207

[ref16] Philips H , Verhoeven V , Morreel S , Colliers A , Remmen R , Coenen S and Van Royen P (2019) Information campaigns and trained triagists may support patients in making an appropriate choice between GP and emergency department. European Journal of General Practice 25, 243–244. 10.1080/13814788.2019.1675630 PMC6853219 31663392

[ref17] Plat FM , Peters YAS , Loots FJ , De Groot CJA , Eckhardt T , Keizer E and Giesen P (2018) Ambulance dispatch versus general practitioner home visit for highly urgent out-of-hours primary care. Family Practice 35, 440–445. 29272417 10.1093/fampra/cmx121

[ref18] Rutten M , Vrielink F , Smits M and Giesen P (2017) Patient and care characteristics of self-referrals treated by the general practitioner cooperative at emergency-care-access-points in the Netherlands. BMC Family Practice 18, 62. 28499354 10.1186/s12875-017-0633-1 PMC5429563

[ref19] Smits M , Hanssen S , Huibers L and Giesen P (2016) Telephone triage in general practices: a written case scenario study in the Netherlands. Scandinavian Journal of Primary Health Care 34, 28–36. 26893132 10.3109/02813432.2016.1144431 PMC4911030

[ref20] Smits M , Rutten M , Keizer E , Wensing M , Westert G and Giesen P (2017) The development and performance of after-hours primary care in the Netherlands: a narrative review. Annals of Internal Medicine 166, 737–742. 28418455 10.7326/M16-2776

[ref21] Uscher-Pines L , Pines J , Kellermann A , Gillen E and Mehrotra A (2013) Emergency department visits for nonurgent conditions: systematic literature review. American Journal of Managed Care 19, 47–59. PMC4156292 23379744

[ref22] Van Den Heede K and Van De Voorde C (2016) Interventions to reduce emergency department utilisation: a review of reviews. Health Policy 120, 1337–1349. 27855964 10.1016/j.healthpol.2016.10.002

[ref23] Van Den Heede KDC , Devriese S , Baier N , Camaly O , Depuijdt E , Geissler A , Ghesquiere A , Misplon S , Quentin W , Van Loon C and Van De Voorde C (2016) Organisatie en financiering van spoeddiensten in België: huidige situatie en opties voor hervorming Health Services Research (HSR). Brussel: Federaal Kenniscentrum voor de Gezondheidszorg (KCE).

[ref24] Van Der Mullen CQH , Van Baelen S , Crits T , Wuyts J and Sabbe M (2017) De patiënt met een niet-planbare zorgvraag naar het gepaste zorgniveau verwijzen: nieuwe 112-1733 geïntegreerde telefonische triage- en regulatieprotocollen. Tijdschrift voor Geneeskunde 73, 241–247

[ref25] Van Der Straten LM , Van Stel HF , Spee FJ , Vreeburg ME , Schrijvers AJ and Sturms LM (2012) Safety and efficiency of triaging low urgent self-referred patients to a general practitioner at an acute care post: an observational study. Emergency Medicine Journal 29, 877–881. 22158535 10.1136/emermed-2011-200539

[ref26] Wheeler SQ , Greenberg ME , Mahlmeister L and Wolfe N (2015) Safety of clinical and non-clinical decision makers in telephone triage: a narrative review. Journal of Telemedicine and Telecare 21, 305–322. 25761468 10.1177/1357633X15571650

[ref27] Young GP , Wagner MB , Kellermann AL , Ellis J and Bouley D (1996) Ambulatory visits to hospital emergency departments. Patterns and reasons for use. 24 hours in the ED study group. Journal of American Medical Association 276, 460–465. 10.1001/jama.276.6.460 8691553

